# The challenge of cultural competence in the workplace: perspectives of healthcare providers

**DOI:** 10.1186/s12913-019-3959-7

**Published:** 2019-02-26

**Authors:** Stephane M. Shepherd, Cynthia Willis-Esqueda, Danielle Newton, Diane Sivasubramaniam, Yin Paradies

**Affiliations:** 1 Centre for Forensic Behavioural Science, Swinburne University of Technology, 1/582 Heidelberg Rd, Alphington, Melbourne, Victoria Australia; 2 Department of Psychology, University of Nebraska-Lincoln, Burnett Hall, Lincoln, NE USA; 3 School of Social & Political Sciences, The University of Melbourne, Gratton Street, Melbourne, Victoria Australia; 4 School of Psychological Sciences, Swinburne University of Technology, John St, Hawthorn, Melbourne, Victoria Australia; 5 Alfred Deakin Research Institute for Citizenship and Globalisation, Deakin University, Burwood, Melbourne, Victoria Australia

**Keywords:** Cultural competence, Cultural safety, Cultural humility, Diversity training, Public health

## Abstract

**Background:**

Cross-cultural educational initiatives for professionals are now commonplace across a variety of sectors including health care. A growing number of studies have attempted to explore the utility of such initiatives on workplace behaviors and client outcomes. Yet few studies have explored how professionals perceive cross-cultural educational models (e.g., cultural awareness, cultural competence) and the extent to which they (and their organizations) execute the principles in practice. In response, this study aimed to explore the general perspectives of health care professionals on culturally competent care, their experiences working with multi-cultural patients, their own levels of cultural competence and the extent to which they believe their workplaces address cross-cultural challenges.

**Methods:**

The perspectives and experiences of a sample of 56 health care professionals across several health care systems from a Mid-Western state in the United States were sourced via a 19-item questionnaire. The questionnaire comprised both open-ended questions and multiple choice items. Percentages across participant responses were calculated for multiple choice items. A thematic analysis of open-ended responses was undertaken to identify dominant themes.

**Results:**

Participants largely expressed confidence in their ability to meet the needs of multi-cultural clientele despite almost half the sample not having undergone formal cross-cultural training. The majority of the sample appeared to view cross-cultural education from a ‘cultural awareness’ perspective - effective cross-cultural care was often defined in terms of possessing useful cultural knowledge (e.g., norms and customs) and facilitating communication (the use of interpreters); in other words, from an immediate practical standpoint. The principles of systemic cross-cultural approaches (e.g., cultural competence, cultural safety) such as a recognition of racism, power imbalances, entrenched majority culture biases and the need for self-reflexivity (awareness of one’s own prejudices) were scarcely acknowledged by study participants.

**Conclusions:**

Findings indicate a need for interventions that acknowledge the value of cultural awareness-based approaches, while also exploring the utility of more comprehensive cultural competence and safety approaches.

## Background

In recent decades, several key public health care reports and research studies on health care experiences have indicated that particular cultural groups are more likely to be underserved, perceive negative treatment, and receive differential treatment outcomes [1–3]. In response, health care systems in North America and other CANZUS nations, have endeavored to adapt their service delivery practices and policies to improve the quality and access of health care to culturally and linguistically diverse groups [4–7]. Industry objectives in health care settings contemporaneously aspire to, i) improve cross-cultural communication ii) enhance responsiveness to the health care needs of diverse patients iii) reduce health care provider discrimination and iv) reduce health care disparities. Health care organizations have embraced and enlisted a variety of cross-cultural educational approaches (e.g., cultural awareness, cultural competence etc.) to achieve these objectives.

Cross-cultural education training for health care professionals is now commonplace and in some settings, mandated [8, 9]. Its principles are often embedded within the strategic plans of health organizations, and human resource departments will often oversee the advancement of such initiatives. Participating health care systems usually employ one, or a combination, of several popular cross-cultural models that have emerged over the past four decades. These include, but are not limited to, cultural awareness, cultural competence, cultural safety, cultural humility and cultural intelligence. There are also multiple off-shoots including anti-racism training and diversity training. The cross-cultural models overlap considerably though they have differing emphases. Cultural awareness focuses on learning about the norms and customs of multi-cultural groups [10]. Cultural safety is concerned with protecting the culture of vulnerable groups by identifying biases and power imbalances within organizational structures [11]. Cultural humility promotes openness and non-judgement while allowing the client to determine how their culture impacts their experiential reality and by extension, the clinical encounter [12]. Cultural intelligence focuses on an individual’s capacity to first recognize and then successfully function in various cultural environments foreign to their own [13]. And cultural competence (though often used generically) is an institutional framework that expands an organization’s internal and external capacity to support and implement protocols that improve worker attitudes, cross-cultural communication, staff diversity, and ongoing relationships with multi-cultural communities and stakeholders [14]. The adoption of these models, or aspects of the models, are believed to ultimately reduce the obstacles that still contribute to the poor health care experiences and unmet health needs of particular cultural minority groups [15].

While the uptake of cross-cultural education models has been widespread across health care systems, evidence for the models’ ability to reduce cross-cultural health care disparities has been slim. There has been some confirmation of temporary improvements in practitioner attitudes and patient experiences post model implementation (usually in the form of a workshop), however the impact on patient treatment outcomes has been largely negligible [16–18]. While there are possible associated explanations for these findings (e.g., model implementation integrity, methodological rigor of validation studies, broader organizational factors) is it uncertain as to why current cross-cultural education strategies have not had stronger effects. In fact, there is evidence indicating that certain delivery styles (e.g., coercive, shame-and-blame) of cross-cultural education may have unintended contrary outcomes for participating staff [8, 19]. Other literature has offered some speculation as to why such trainings and associated initiatives may fall short in achieving anticipated outcomes in health care settings [20–23]. Shepherd [9] points to the impracticality of retaining and then utilizing ‘quickly-learned’ cultural knowledges, customs and interaction styles beyond cross-cultural workshops. The potential for essentializing and/or homogenizing cultural groups leading to stereotypical pre-conceptions has also been referred to [22, 24–26]. Moreover, numerous factors may impact a cross-cultural clinical encounter beyond cultural differences (e.g., personality, temperament, cognitive ability, level of education, socio-economic status, mental health, universally poor service delivery). It is clear that further research efforts are required to explore how cross-cultural education is typically delivered, received and implemented in various health care settings.

One way to acquire this information is to ascertain the views and experiences of health care professionals themselves. Cross-cultural training workshops are often validated by health care professionals via pre-post intervention surveys. However, the insights and perceptions of health care professionals are infrequently sought when attempting to develop, define or refine effective cross-cultural practice. Few studies globally, have explored the perceptions of professionals on cross-cultural education initiatives in health care. A number of studies from Australia (samples < *n* = 20) explored the barriers to effective cross-cultural communication as identified by health workers and the importance they afforded to cultural competence [27–30]. Similar studies were conducted with small samples of nurses in Scotland [31] and Ireland [32]. In North America, a study from Canada assessed attitudes towards cultural competence for 170 registered nurses [33] and a study from USA explored how 31 public health nurses gauged their own levels of cultural competence and experiences of culturally competent care [34]. The opinions of both health care providers and medical staff on some of the challenges faced when working with diverse clientele, were obtained in the context of broader studies on cross-cultural health care in the USA [35] and Sweden [36]. Furthermore, health care professionals’ perceptions of their own cultural competence has been examined across professions (i.e., physicians compared to nurses) [37] and in relation to their alignment with patient observations [38]. Overall, the above literature illustrates that health care professionals, despite possessing varying levels of cross-cultural knowledge, largely acknowledge the importance of cross-cultural awareness and demonstrate a willingness to improve their cross-cultural communication skills. Language barriers, low client health literacy and bureaucratic constraints are regularly offered as barriers to effective cross-cultural service delivery [27–30]. It is clear however, that further research is warranted in this space to acquire a more nuanced understanding of how health care providers and professionals view and experience intercultural encounters, and the educational initiatives implemented to enhance such encounters. In response, this study aims to gather and explore i) the general perspectives of health care professionals on culturally competent care, ii) their experiences working with multi-cultural patients, iii) their perceptions of their own levels of cultural competence and iv) the extent to which they believe their respective organizations address cross-cultural challenges in the workplace. Professionals were recruited from health care systems in a Mid-Western region of the USA where no prior research of this nature has been conducted. We anticipated that the majority of professionals will value the importance of cross-cultural education and training but may differ with regards to both their own and their organization’s perceived knowledge, and which aspects of cross-cultural education they believe are relevant to their practice.

## Methods

### Participants

Participants were recruited across three major health care systems and one university student health center from a Mid-Western state in America. The first health system, a major university medical training facility comprises a network of two hospitals and 40 clinics. The second health system, is a state-run network of over 30 major hospitals and clinics. The third, is a regional faith-based network of 14 hospitals and over 400 clinics. The university student health center provides a range of medical services on a major university campus. Combined, the three health systems and university student health center, operate the majority of medical facilities in the state.

### Materials

Participants completed a 19-item questionnaire ascertaining their general perspectives on culturally competent care, their experiences working with multi-cultural patients, their own levels of cultural competence and the extent to which they believe their organizations addressed cultural competence in the workplace. The questions were both multiple choice (*n* = 15) and open-ended (*n* = 4). For the multiple choice questions, participants were asked the extent to which they agree or disagree with various general statements (e.g., Do you think health service providers should consider a patient’s cultural background when treating them?), to reflect on how often their own skills align with best practice cross-cultural care (e.g., Do you feel that you attend to the cultural needs of patients from difference cultural backgrounds?) and the importance they afford to various skillsets (Do you think it is important to learn about different cultures as part of your practice?). Open ended questions prompted participants to elaborate on their perceptions and experiences (e.g., What areas of cultural awareness/competence do you feel that you and/or your organisation perform well?). The combination of both closed and open-ended questions allowed for a baseline understanding of participant views across key ideas, which could then elucidated through nuanced personal narratives.

The questionnaire was developed by the study authors through their own expertise and after reviewing the relevant public health and cross-cultural health care literature. Questions were selected based on their relevance to the study (e.g., health care environments) and their alignment with the expectations and principles of cross-cultural communication philosophies (e.g., cultural competence, cultural awareness, cultural safety). No previous validated questionnaire specifically tailored to the cross-cultural experiences of health care professionals was located for use.

### Procedure

Researchers contacted participating health care networks in mid-2016 to ascertain their interest in being involved in the study. After receiving support from their respective internal review boards, the organizations distributed an online link to the study survey to clinical and professional staff via email. The link was accompanied with a short passage enquiring if staff were interested in participating in an anonymous online research study survey on cross-cultural health care service delivery experiences.

Individuals who opted to click on the study link were presented with a digital consent form outlining the requirements and their involvement in the study. After consenting, participants then completed the anonymous online health care provider cross-cultural experiences survey. The questionnaire was conducted on an online research survey software program. The duration of participation ranged from 10 to 30 min. All participants received a $10 online gift voucher for their contribution. Data was collected across the months of August and September in 2016. It is unknown as to exactly how many staff at each organization received the study recruitment email.

### Data analyses

A mixed methods study design was employed. First, percentages across participant responses were calculated for all multiple choice items. Second, the four open-ended questions were then qualitatively analysed. After extracting the data from the online research survey program, a thematic analysis was undertaken by a primary coder (author DN) using a progressive process of classifying, comparing, grouping and refining groups of text segments to create and then clarify the definition of categories, or themes, within the data [39]. For the purposes of reliability, another coder (author SS) independently coded a subsection of interview notes and cross-checked these with the findings of the primary coder. Following coding, dominant themes were cross-checked between raters until a consensus was reached. Participant percentages across categories for the 19 multiple choice questions were calculated and tabled.

## Results

Data were collected from a total of 56 health care workers. Four additional individuals elected not to consent to participate in the study after viewing the information statement and consent form page. Due to the anonymous nature of the study, the distribution of participants across the four health care organizations was unknown. The mean age of the sample was 38.66 (SD: 12.03, range 20–65) years. The majority were female (*n* = 52, 92.9%). Over 85% (*n* = 48) of the sample identified as White/Caucasian, with 8.9% (*n* = 5) identifying as Hispanic/Latino, 3.6% (*n* = 2) identifying as Middle Eastern and 1.8% (*n* = 1) identifying as African-American. The self-described professions of the participants included Nurse/Nurse Practitioner (*n* = 18, 32.1%), Mental Health Professionals (*n* = 9, 16.1%), Medical Assistants (*n* = 7, 12.5%), Hospital/Clinic Administrators (n = 7, 12.5%), Medical Receptionist (n = 5, 8.9%), Physicians (*n* = 3, 5.4%), Physiotherapists (*n* = 2, 3.6%), Interpreters (n = 2, 3.6%), Pharmacist (n = 1, 1.8%), Community Support Worker (n = 1, 1.8%), Medical Lab Technician (n = 1, 1.8%). Regarding years of experience in their current profession, 26.8% (*n* = 15) of the sample reported more than 20 years of experience, 23.2% (*n* = 13) reported between 11 and 20 years of experience, 21.4% (*n* = 12) reported between 5 and 10 years of experience, and 28.6% (*n* = 16) reported less than 5 years of experience.

Participant perceptions on the importance of cross-cultural education and communication in health care settings are presented in Table 1. The vast majority of participants clearly believed that cultural considerations are an important component of best practice health care and that professionals should be learning about different cultural groups. Responses varied as to whether one’s organization should be making improved efforts to meet the needs of diverse clientele and whether cultural-specific approaches to health (e.g., traditional/spiritual healers) should be accommodated.

**Table 1:** Cross-cultural perceptions of health care professionals (Agree – Disagree)

Health care provider questions	Strongly Agree % (*n*)	Agree	Neither Agree/ Disagree	Disagree	Strongly Disagree
Do you think health service providers should consider a patient’s cultural background when treating them?	60.7 (34)	28.6 (16)	3.6 (2)	5.4 (3)	1.8 (1)
Cultural awareness is important in providing best-practice health care.	75.0 (42)	23.2 (13)	1.8 (1)	–	–
Being able to effectively communicate cross-culturally with patients is important to best practice health care.	83.9 (47)	16.1 (9)	–	–	–
Do you think it is important to learn about different cultures as part of your practice?	75.0 (42)	21.4 (12)	3.6 (2)	–	–
Do you think learning about different cultures improves service delivery with multi-cultural patients?	75.0 (42)	21.4 (12)	3.6 (2)	–	–
Do you feel that your organization could do a better job at accommodating the needs of patients from diverse cultures?	8.9 (5)	35.7 (20)	35.7 (20)	16.1 (9)	3.6 (2)
Do you think other cultural models of health are useful to complement conventional health care approaches?	17.9 (10)	35.7 (20)	35.7 (20)	10.7 (6)	–

*n* = 56

Participants were also asked to designate the level of perceived importance they afforded to having staff from diverse cultural backgrounds represented at their workplace. Almost three-fifths (39.3%, *n* = 22) of the sample believed staff diversity to be ‘extremely important’, 28.6% (*n* = 16) believed staff diversity to be ‘important’ and a further 12.5% (*n* = 7), 17.9% (*n* = 10), 1.8% (*n* = 1), believed staff diversity to be moderately, slightly, or not at all important, respectively. Most participants (91.1%, *n =* 51) acknowledged that bi-lingual staff worked at their organization. Three participants (5.4%) did not provide an answer to this question.

Participant perceptions of their own cross-cultural awareness experiences and capabilities are presented in Table 2. Health care providers acknowledged that they regularly treat patients of color and that they would commonly attend to their needs. The majority also noted that sometimes, and for some, often, it was more challenging to treat or engage with patients from a different cultural background to their own. Over 40% (*n* = 23) of participants believed that their cultural background often makes patients from other cultural backgrounds uncomfortable. Moreover one-fifth (*n* = 12) of the sample thought that their cultural background sometimes made patients of color feel anxious or nervous. Participants were also asked to describe how satisfied they were with their own perceived level of cross-cultural knowledge. Under 15% (*n* = 8) reported that they were ‘extremely satisfied’ with their level of knowledge, 64.3% (*n* = 36) were ‘satisfied’ with their level of knowledge, 16.1% (*n* = 9) were neither dissatisfied or satisfied, and 3.6% (*n* = 2) were ‘dissatisfied’. No participant reported ‘extreme dissatisfaction’ with their level of cross-cultural knowledge.

**Table 2:** Cross-cultural perceptions of health care professionals (Always – Never)

Health care provider questions	Always % (*n*)	Often	Sometimes	Rarely	Never
Do you feel that you attend to the cultural needs of patients from different cultural .backgrounds	39.3 (22)	55.4 (31)	5.4 (3)	–	–
How often do you treat patients of color?	80.04 (45)	16.1 (9)	3.6 (2)	–	–
Is it more difficult to engage with/treat people from a different culture to your own?	–	21.4 (12)	62.5 (35)	14.3 (8)	1.8 (1)
Do you think your cultural background makes some patients from different cultural backgrounds uncomfortable?	1.8 (1)	39.3 (22)	–	41.1 (22)	17.9 (10)
Do you think that some patients of color feel anxious/nervous around you during treatment?	–	1.8 (1)	19.6 (11)	42.9 (24)	35.7 (20)

*n = 56*

### Thematic analysis

Participants responded to four open-ended questions on cross-cultural health care delivery. They were asked to reflect on ways to improve cross-cultural health care and the extent to which they perceived their organizations to be suitably performing in this area. Each question and the associated response themes are presented below.

### What factors/skills do you think could improve cross-cultural health care?

#### Education/training

Many (*n* = 31) participants believed that formal cross-cultural education and training would indeed improve their organization’s capacity to provide cross-cultural health care. Most of the suggestions were oriented to learning about multi-cultural customs.

*“Knowledge regarding different cultures and customs”.* (Registered Nurse).

*“Educational lecturers and/or classes that can assist others in learning about diverse cultures”.* (Community Support Worker).

Some felt that this education should be mandatory or at least regularly provided to staff.

*“Regular educational seminars/courses on addressing different cultures in health care settings”.* (Registered Nurse).

Two participants felt that education and training should have a particular focus on systemic issues experienced by patients from minority backgrounds.

*“Trainings of awareness, bias and privilege”.* (Mental Health Professional).

*“Increased education regarding institutional inequality on the local, state, and national levels”.* (Mental Health Professional).

#### Interpreter services

The second most common response theme was having interpreter services available and accessible to all patients.

*“Interpretation services throughout all clinics in the area”.* (Medical Assistant).

Some participants also suggested that that there should be more bi-lingual professionals.

*“More providers who are bilingual or who offer services in languages other than English”.* (Mental Health Professional).

#### Diversification of staff

One participant expressed that organizations should aim for cultural diversity when recruiting staff.

*“Increased recruitment and hiring of professionals who are people of color and people of other-than-white cultural identities and experiences”.* (Mental Health Professional).

### What areas of cultural awareness/cultural competence do you feel that you or your organization perform well?

#### Access to interpreters

Nineteen participants commented positively on the provision of interpreters to patients requiring language assistance. *“We provide free interpreters for any clients who need one”* (Hospital/Clinic Administrator)*. “I love that we have interpreters here”* (Registered Nurse)*. “We provide on-site interpreters to translate for the patients to provide the best quality care”.* (Interpreter).

#### Cultural awareness/sensitivity

Eleven participants believed that their organization was culturally aware, respectful of cultural customs and mindful of the specific needs of patients from different cultural backgrounds*. “We are sensitive to everyone’s needs and go over and beyond to meet those expectations”* (Medical Receptionist). *“There is a general cultural expectation in the organization that we are welcoming and respectful of all kinds of people”* (Mental Health Professional)*. “We try to be accommodating when at all possible regarding cultural customs”* (Hospital/Clinic Administrator).

#### Education/training

A number of participants reported that their organization encouraged a commitment to cross-cultural training. This was evidenced by cross-cultural training undertaken by staff. *“Having training to make us aware of cultural beliefs”* (Mental Health Professional)*. “Education about our population (catchment area) was provided so providers and staff had some background and understanding”* (Registered Nurse)*. “Monthly diversity trainings”* (Mental Health Professional).

#### Culturally diverse staff members

Five participants believed that their organization benefitted from hiring diverse staff members. This was viewed as being of great value to the multi-cultural patients attending their organization. *“Our organization does an excellent job of hiring diverse staff members, for the most part, especially in our refugee services and support staff positions”* (Mental Health Professional)*. “Having providers and staff with different cultural backgrounds has been useful”* (Hospital/Clinic Administrator).

#### Assessment of needs/rapport development

Some participants underscored the benefits of directly asking patients about their cultural needs and how best they could be accommodated. *“Asking how we can best care for their cultural needs at first appointment”* (Registered Nurse)*.* Other participants reported that professionals at their organization spent some time ‘getting to know’ patients from different cultural backgrounds in order to establish a relationship of trust. *“We treat a lot of different cultures and we ask questions sometimes in order to get to know them…culturally some people need more time with a doctor than others which can make it difficult but can also be beneficial in creating a bond”* (Interpreter).

#### Resources

Four participants commented positively on their organization’s provision of cross-cultural educational resources for both patients - *“We have information printed in different languages for communication of medical information”* (Physician), and staff - *“Education and resources related to all cultures available to staff”* (Registered Nurse).

### What was the nature of the training?

This open-ended question followed the initial yes/no question, “Did your health care training include a cultural awareness/competence component”, to which 57.1% (*n* = 32) participants affirmed that they had received some form of cultural training as part of their professional education. The thematic responses from these 32 participants are illustrated below.

#### Format

Ten participants indicated that the training they had undertaken was online. A smaller number of participants stated that cross-cultural training was conveyed through presentations from guest speakers and a further two participants claimed that their training was obtained through clinical experience. The majority of participants stated that the training was a requirement for all staff members. *“The cultural competence training is an online course which all staff must complete when they are first hired, and then annually afterwards”* (Mental Health Professional)*. “Required for licensing”* (Mental Health Professional)*.*

#### Content

Participants described the content of the training as primarily focused on *“cultural norms and differences”* (Nurse Practitioner, Physiotherapist).

### How would you improve the cultural competence of your organization?

#### Further education/training

Most participants believed that cultural competence training should be *“regular”* (Interpreter) and *“mandatory”* (Registered Nurse)*.* More specifically, several participants suggested that training should involve *“speakers from different cultural backgrounds”* (Hospital/Clinic Administrator)*. “It would be helpful to have training/discussions with people from different cultures/ethnic backgrounds who are willing to meet with health care staff in order to learn about their cultures/norms and how health care providers would be most effective in helping those clients”* (Nurse Practitioner). Some participants advocated for regular organizational-wide meetings for staff to have discussions about cultural competence. *“Talking about the problems we may encounter based on culture”* (Interpreter)*.* Others felt that any cultural education should only focus on the cultural groups within the organization’s catchment area. *“Education in regards to the geographical location of the patients we provide health service to”* (Hospital/Clinic Administrator)*. “More detailed training on the specific cultures that are prevalent in the area rather than presenting it as more global”* (Registered Nurse)*.* Only one participant referred to discriminatory behaviors. *“Increase accountability for micro-aggressions, institutional inequality, racism, sexism etc. with the understanding that we all have these issues and the most important thing is to be aware of them and work to challenge them within ourselves”* (Mental Health Professional).

#### Staff diversity

Although few participants commented on staff diversity, disagreement was evident. Three participants believed that their organization would be more culturally competent if there was greater cultural diversity among staff members. *“Increase the number of staff members of color and staff members who are multi-lingual”* (Mental Health Professional). In contrast, two participants stated that cultural background was not the main priority when hiring new staff members. *“I hire the best person for the job, not for their cultural background”* (Hospital/Clinic Administrator).

#### Already competent

Several participants believed that their respective organizations were already meeting cultural competence principles. *“My clinic provides excellent services and a willingness to diversify amongst multiple different cultures”* (Medical Assistant)*. “I feel we provide all cultures with adequate care”* (Physician)*.*

## Discussion

Cross-cultural educational initiatives for professionals are now commonplace across a variety of sectors including health care. A growing number of studies have attempted to explore the utility of such initiatives on workplace behaviors and client outcomes. Yet few studies have explored how professionals perceive cross-cultural educational models (e.g., cultural awareness, cultural competence) and the extent to which they (and their organizations) execute the principles in practice. This study aimed to address this gap in the literature by gathering the perspectives of a sample of health care workers from a Mid-Western state in the United States of America. The insights gained from the research provide a useful contribution to the practical literature on cross-cultural professional training. It is important to ascertain the attitudes and professional experiences of health workers when working cross-culturally, to assist in developing functional and effective trainings that are endorsed by the very professionals that they are designed for.

Like prior research [29, 31, 33, 35], the vast majority of the sample acknowledged that a consideration of a client’s culture was of importance. Possessing cultural knowledge was widely perceived to be ‘best practice’ and necessary for effective cross-cultural communication and service delivery. Half the sample agreed that alternative cultural models of health would augment existing approaches to care. There is a wide body of literature illustrating culturally bound models of health and symptom expression styles that are believed to deviate from, or partially overlap with western diagnostic categories [40, 41]. Other participants may have been unaware of the differing concepts of health and wellbeing possessed by particular cultural groups. Some may also have perceived that certain cultural or ‘folk’ health beliefs clash with conventional methods to the detriment of their patient, a view documented in prior research with health professionals [27–29, 42]. Nonetheless, approximately 95% of the sample believed that they always or often attend to the cultural needs of their patients. This near consensus arose despite over 80% of participants sharing that they often or sometimes found it more difficult to engage with or treat patients from cultures different to their own. Additionally, almost 60% acknowledged that their own culture may make some patients uncomfortable. The sample’s apparent confidence in their own abilities to work effectively cross-culturally despite obvious challenges may reflect workplaces committed to cultural competence initiatives and diversity. Some participants in this study may also genuinely be equipped with cultural knowledge and the facility to recognize the limitations of their own knowledge when working with different cultures as per the cultural humility model. However, there is also a possible incongruity between the assumption that one is commonly addressing cultural needs and the frequent experiencing of challenges when working with minority patients, which may signal an over-confidence and/or unawareness. This is often referred to in the cultural safety literature as a failure to interrogate one’s own cultural beliefs, and how a vocation may have built-in entrenched dominant culture norms and standards that impact cross-cultural clinical encounters [43]. It was unknown as to how participants discerned that their cultural background had made their patients uncomfortable. They may have been acutely aware of how the dominant culture is viewed through the lens of historically oppressed minority patients. Alternatively, they may have received negative feedback or complaints from patients because of their actions.

In a similar pattern to the above findings, just over half of the sample (57%) had received some form of cross-cultural training as part of their overall health care education, yet almost 80% were satisfied with their level cultural knowledge. The cross-cultural training undertaken, was largely described as learning about the norms and customs of other cultural groups (either online or through presentations) which is reminiscent of the cultural awareness model. Alternative cross-cultural models (cultural humility, cultural safety) critique the cultural awareness approach for homogenizing cultural groups, and effectively ‘trading in stereotypes’ [12, 44]. A possible discrepancy between real and perceived cross-cultural knowledge is conceivable here.

Participants were asked to outline what areas of cross-cultural care they believed that they (or their organization) perform well. Answers here, would perhaps illuminate the apparent confidence many participants possessed when working cross-culturally despite a significant minority not having undertaken formal cross-cultural training. The availability of interpreters was the most common example of effective cross-cultural care as denoted by participants. A lack of interpreter services has been identified as a common obstacle for cross-cultural service delivery in prior studies [27, 29, 30, 34]. There was also a belief from numerous participants, that their respective organizations were culturally respectful and attentive to the cultural needs of clients. Several participants stated that their organizations offer cultural training (some on a regular basis), employ multi-cultural staff, and provide educational resources for staff and patients. Staff diversity was emphasized as beneficial for multi-cultural patients. Perhaps this was in regard to their capacity to speak languages other than English – over 90% of participants indicated that they work alongside bi-lingual staff. It is possible from the above testimony that many participants work in organizations that possess some of the attributes allied with the cultural competence model. Cultural competence is an organizational-wide approach to enhancing effective cross-cultural communication which includes a number of interconnecting initiatives (e.g., staff diversity, staff training, interpreter services, improving staff attitudes to cross-cultural care) [15]. Such a framework could potentially engender a safe multi-cultural working environment despite staff not having undergone direct cultural training themselves. In contrast, it is perhaps more likely that participants viewed effective cross-cultural care as essentially a communication matter as opposed to a multi-faceted institutional framework. Rather than perceiving cross-cultural care as a broader phenomenon encompassing power imbalances, dominant culture biases and contrasting worldviews (interpretations seldom articulated by the sample), most participants may have simply regarded cross-cultural care from an immediate practical standpoint whereby interactive/informational barriers require alleviation; hence the highlighting of interpreter services, staff bi-linguicism and assessment rapport development. Starr and Wallace [34] found that the availability (or lack of) interpreters and gender-specific providers were some of the most commonly raised themes in a sample of American public health nurses when discussing culturally competent care. Only a small number of participants referred to diversity hiring strategies directly. A tension between affirmative action and colour-blind approaches was evident among some of the responders.

Participants were asked to identify which factors/attributes improve cross-cultural care, and more pointedly, to offer recommendations to improve the cultural competence of their own organizations. Two main themes were canvassed in relation to the first question. Participants cited the need for ongoing cross-cultural education and training in the workplace. Responses again appeared to denote the cultural awareness approach – a focus on cultural norms, customs and health beliefs - which is the most common form of cross-cultural education universally, yet the most criticized in the literature. Only two participants described the need for training to encompass themes of systemic bias and privilege which reflect the cultural safety approach. Cross-cultural education was the leading recommendation among the sample for improving organizational cultural care. A desire for regular staff meetings on cross-cultural issues was posited. There was also a preference among several respondents to focus attention on the cultural needs of clientele within the organization’s catchment area, a recommendation proffered in previous research. No participants referred to any bureaucratic procedures that constrained their ability to work effectively cross-culturally, a finding noted in several prior studies [27, 29, 30]. Somewhat surprisingly, several participants disclosed that their organizations were already culturally competent and as such, did not offer any recommendations to improve cross-cultural care. Cultural competence is often described by its proponents as an evolving process rather than a ‘clear-cut’ actuality [45]. There is some evidence however from the same mid-western region that multi-cultural patients are generally satisfied with their treatment from health care providers. Shepherd et al. [46] found that racial minorities from a mid-Western state reported that they had good access to health care services, were not afraid to visit mainstream medical services and experienced low levels of racism and poor treatment.

### Implications

Findings from the study should be considered in light of several limitations. First, caution is advised when generalizing results beyond Mid-Western health care settings. Second, the sample, like previous cohorts in the extant literature, was predominantly female and white/Caucasian. Prior research has suggested that female health care staff are more likely to possess patient-centered communication styles [47, 48] which may be more conducive to cross-cultural care. Ohana & Mash [38] discovered that discrepancies between physicians’ perceptions of their own cultural competence and their patients’ perceptions are reduced if the physician is female. Moreover, few cultural minorities participated, which may reflect the demographics of health care professionals in the region, and more broadly, the state population. Health professionals from cultural minority backgrounds may be more cognisant of the systemic and/or historical challenges faced by minority patients and may therefore be inclined to support broader cross-cultural educational approaches that address explicit/implicit discrimination, power structures and historical injustices [11, 12, 49–51]. Furthermore, the exact response rate in the study was unknown – health systems were unable to disclose how many study invitations were distributed across their respective networks. Third, no direct patient data was collected to corroborate the cohort’s generally optimistic assertions that they and their organizations provide care that is meeting the needs of their multi-cultural clientele. Prior research has found a weak relationship between medical professionals’ perceptions of their own cultural competence and their patient’s perceptions of their cultural competence [38]. Last, the terminology on the questionnaire (i.e., ‘diverse cultures’, ‘cultural background’ etc.) was left open to interpretation. It is possible that these broad descriptors may have influenced responses.

The health workers in our sample appeared to view cross-cultural education from a ‘cultural awareness’ perspective. Effective cross-cultural care was often defined in terms of cultural knowledge (e.g., norms and customs) and facilitating communication (the use of interpreters). A dearth of respondents referred to broader, systemic components of cross-cultural care such as a recognition of racism (explicit and implicit), power imbalances, entrenched majority culture biases and the need for self-reflexivity (awareness of one’s own prejudices). Although a number of participants supported workplace diversity, this appeared to be in the course of improved communication with minority patients rather than advocating for diversity per se, or for cultural diversity at the executive, or ‘decision-making’ levels. As speculated earlier, cross-cultural education was perhaps viewed as an immediately applied or ‘hands on’ phenomenon rather than a holistic, structural approach. Prior research has found that clinicians have a preference for ‘active behavioural simulations’ as a cross-cultural training method [52].

While most models of cross-cultural education encompass learning about the ‘other’ to some degree, simply absorbing the health beliefs, idiosyncrasies and traditions of particular cultural groups is somewhat superficial and as such, unlikely to advance cross-cultural communication [9, 44]. Advocates of later cross-cultural models (cultural competence, cultural humility, cultural safety) would indeed stress the need to augment the cultural awareness styled training undertaken and demanded by participants in the study. The compelled awareness of one’s own biases and their institutions’ potential to marginalize patients from non-mainstream cultural groups would be likely additions to teachings. In fact proponents of later models may argue that the over-confidence displayed by some participants in our study could be a reflection of their inability to recognize their own limitations and prejudices – and as such, underscoring the need for participants to undergo broader, holistic training. Intuitively, there is merit to multi-faceted models that incorporate socio-historical and political factors. At the same time, robust evidence for cross-cultural educational models, regardless of their content, is meagre [9, 44, 53–55]. Interpreter and bi-lingual health worker services possess some empirical support [56–58] though further research is warranted [17]. Thus, a conundrum unfolds whereby participants appear to be content with a limited model (in cultural awareness) yet the more expansive, recommended alternatives (cultural safety etc.) have yet to demonstrate rigorous utility beyond anecdotal evidence. The potential consequences of politically charged ‘blame and shame’ approaches (which are more likely to occur within cultural safety-styled trainings) have also been documented [8, 19]. Further research is required to rigorously test the validity of the various models and their specific assumptions. It is also important to ensure that cross-cultural models are relevant to specific workplaces, flexible enough to address immediate challenges identified by frontline staff, and seek realistic, practical goals that are clear, quantifiable and have evidence for their utility.

## Conclusions

This study finds that health care professionals from a Mid-Western region of the USA value the possession and pursuit of cultural knowledge when working with multi-cultural populations. Practical cross-cultural approaches endorsed by staff (e.g., interpreter services) appeared to be enthusiastically supported and were thought to be associated with effectual cross-cultural care. However, the principles of systemic cross-cultural approaches (e.g., cultural competence, cultural safety) were scarcely acknowledged by study participants. The findings indicate a need for interventions that acknowledge the value of cultural awareness-based approaches, while also exploring the utility of more comprehensive cultural competence and safety approaches.

## Abbreviations

CANZUS: Canada, Australia, New Zealand, United States
USA: United States of America
